# Spatial Studies in Augmented, Virtual, and Mixed Reality: Potentials, Challenges, and Requirements

**DOI:** 10.1007/s42489-026-00219-2

**Published:** 2026-05-07

**Authors:** Julian Keil, Annika Korte, Dennis Edler, Frank Dickmann

**Affiliations:** Geography Department, Ruhr University Bochum, Bochum, Germany

**Keywords:** Spatial research, VR hardware, Extended reality, Immersion, Experiment design, Räumliche Forschung, VR Hardware, Erweiterte Realität, Immersion, Versuchsaufbau

## Abstract

The use of extended reality (XR) hardware for virtual reality (VR) and augmented reality (AR) applications provides new opportunities for innovative spatial research. It enables ways for immersive (re-)presentation of and interaction with spatial environments that are not possible with monitor-based studies. Simultaneously, using XR hardware can either extend opportunities of studies in physical material environments with virtual augmentations or provide extensive experimental control over stimuli and distractors by exposing participants to fully virtual spatial representations. However, these new research approaches require adjustments to the planning and execution of spatial studies. Furthermore, new challenges such as the occurrence of VR sickness or the need for accurate spatial tracking under different environmental conditions must be addressed. In this article, we examine basic potentials, challenges, and requirements associated with spatial research with XR hardware. Furthermore, we address inherent trade-offs of various design decisions, such as the selection of a specific head-mounted display (HMD), software tools, and general experiment design characteristics such as available locomotion methods and stimulus presentation. By providing an overview of important issues associated with the design of spatial studies with XR hardware, we aim to give spatial researchers an entry point into the development of VR and AR studies, or to offer additional food for thought for researchers with initial XR experience.

## Abstract (de)

Der Einsatz von Extended Reality (XR) Hardware für Virtual Reality (VR) und Augmented Reality (AR) Anwendungen eröffnet neue Möglichkeiten für innovative Raumforschung. Sie ermöglicht immersive Darstellungs- und Interaktionsformen räumlicher Umgebungen, die mit monitorbasierten Studien nicht realisierbar sind. Gleichzeitig kann XR-Hardware entweder die Möglichkeiten von Studien in realen Umgebungen durch die Anreicherung mit virtuellen Stimuli erweitern oder eine umfangreiche experimentelle Kontrolle über Stimuli und Störfaktoren gewährleisten, indem Probanden vollständig virtuellen Raumdarstellungen ausgesetzt werden. Diese neuen Forschungsansätze erfordern jedoch Anpassungen der Planung und Durchführung von Raumstudien. Darüber hinaus müssen neue Herausforderungen wie VR-Sickness oder die Notwendigkeit einer präzisen räumlichen Erfassung unter verschiedenen Umgebungsbedingungen bewältigt werden. In diesem Manuskript untersuchen wir die grundlegenden Potenziale, Herausforderungen und Anforderungen der Raumforschung mit XR-Hardware. Außerdem gehen wir auf die inhärenten Kompromisse verschiedener Designentscheidungen ein, wie beispielsweise die Auswahl eines spezifischen Head-Mounted-Displays (HMD), von Software-Tools und allgemeinen Merkmalen des Versuchsaufbaus, wie verfügbare Fortbewegungsmethoden und die Stimuluspräsentation. Mit der Bereitstellung eines Überblicks über wichtige Aspekte im Zusammenhang mit der Konzeption räumlicher Studien mit XR-Hardware möchten wir räumlichen Forschern einen Einstieg in die Entwicklung von VR- und AR-Studien ermöglichen oder Forschern mit ersten XR-Erfahrungen zusätzliche Anregungen bieten.

## Introduction

The launch of innovative virtual reality (VR) and augmented reality (AR) hardware in recent years has provided new research opportunities for cognitive and spatial research. Fully virtual environments or a mixture of virtual and physical material elements can be visually presented and explored in ways that closely resemble the natural perception of physical material (“real”) environments (Hruby et al. 2020a). Participants can explore these environments using either natural or (in the case of fully virtual environments) artificial locomotion and perceive spatial characteristics from different visual perspectives (Keil et al. 2021b; Kelly et al. 2020).

The use of virtual elements allows researchers to expose participants to spatial characteristics or complete environments that otherwise could not be experienced due to reasons such as remoteness, financial constraints, safety concerns (Liang et al. 2019), or the fact that they no longer exist or have not yet been built (Keil et al. 2021b). Examples are spatial planning scenarios (Jamei et al. 2017), reconstructions of historical sites (Edler et al. 2019a; Kersten et al. 2018; Walmsley and Kersten 2020), education (Edler et al. 2025), or emergency training and simulation (Guo et al. 2020). Furthermore, compared to experiments conducted in physical material environments, VR-based experiments allow for greater standardization and the systematic elimination of potential distractors (Keil et al. 2021b; Pan and Hamilton 2018). Providing only virtual visual and auditory input allows experimenters to fully control parameters such as lighting conditions, ambient noise, or the appearance of passers-by. This makes it possible to isolate and investigate specific spatial elements and their effects on spatial memory, perception, and behavior (Keil et al. 2024; Shi et al. 2021; Zucchelli et al. 2021), or even investigate physiological and neurological responses associated with the perception of these spatial elements (Chen et al. 2017; Dickmann et al. 2024; Hofmann et al. 2021; Keil et al. 2023; Reichert et al. 2026).

By exposing participants to partially or fully virtual environments using immersive AR or VR hardware, a feeling of presence can be evoked (Edler et al. 2018; Hruby et al. 2020b). This sense of presence is associated with the illusion that the environment depicted is actually visited and not just viewed through screen(s) (Slater et al. 1996; Waterworth and Riva 2014). It encourages participants to behave in ways that approximate real-world behavior despite their awareness that the perceived stimuli are simulated (Slater and Sanchez-Vives 2016). Thus, opposed to less immersive ways to display virtual spatial environments (e.g., via 2D monitors), using immersive VR or AR hardware allows us to obtain more ecologically valid findings of spatial perception and behavior in partially or fully virtual environments (Pan and Hamilton 2018).

In summary, using VR and AR hardware can greatly expand the possibilities for spatial and cognitive scientists to investigate spatial phenomena. It allows to simulate environments that are not accessible in physical material space. At the same time, the spatial perception and behavior of the participants remain realistic and ecologically valid. However, in comparison with traditional cognitive and spatial studies that either present “natural” physical material environments or display stimuli using 2D monitors, using VR or AR techniques also introduces new challenges and requirements. In this article, we aim to address these challenges and requirements, highlight unique characteristics of VR- and AR-based research, and provide food for thought for new research approaches. The topics covered here are sorted into three main categories: hardware, software, and experiment design. They can act as an initial guideline for researchers who are new to the use of VR and AR or provide additional guidance for researchers who already use VR or AR hardware in their cognitive and spatial studies. The aim of this article is to provide a structured methodological overview of the central potentials, challenges, and practical requirements associated with XR-based spatial research.

## Hardware

In the past, VR hardware and AR hardware were often custom-made, expensive, and very limited in their capabilities. This radically changed with the introduction of the Oculus Rift, the HTC Vive (both VR devices), and the Microsoft HoloLens (AR device). The introduction of these devices was followed by a wave of (relatively) affordable VR and AR hardware releases. Today, there is a wide selection of devices for researchers to choose from. In this section, we will address how the selection of specific devices and their characteristics affects the immersion of displayed virtual environments or elements as well as the available options for experiment design. Furthermore, remaining limitations of modern VR and AR hardware are discussed.

### Virtual Reality, Augmented Reality, Mixed Reality, Extended Reality?

Head-mounted displays (HMDs) used to display virtual elements are often characterized as VR or AR HMDs. These characterizations are based on the reality–virtuality continuum introduced by Milgram et al. (1995). The continuum ranges from purely real environments to purely virtual environments.

On the virtuality–reality continuum, AR can be located close to real environments. It describes an overlay of digital (visual or auditory) information “on top of the physical world” (Noor 2016). In the totality of the perceived spatial stimuli, the physical material stimuli predominate. On the other hand, VR is more difficult to define. It is sometimes described as a completely synthetic world (Milgram et al. 1995), thus being located on the opposite (purely virtual) end of the reality–virtuality continuum. However, HMDs defined as VR devices still rely on input from the physical world, such as head and body movements, which are mirrored in the virtual environment. This absoluteness in the definition of VR as completely synthetic is therefore controversial.

Some manufacturers avoid using the terms AR and VR entirely and instead refer to their HMDs as mixed reality (MR) devices. This is especially the case since the introduction of pass-through devices such as the HTC Vive Pro. These devices have the additional function to send camera recordings of the physical material surroundings onto screens in the HMD and augment these recordings with virtual elements. Thus, MR devices can either display only the recordings of the surroundings, only virtual stimuli, or any desired combination of both. Therefore, they cannot be assigned to a fixed location on the virtuality–reality continuum described by Milgram et al. (1995).

A commonly used umbrella term intended to encompass AR, VR, and MR is “extended reality” (XR). This term facilitates the classification of concepts that apply to VR and AR as well as to MR hardware and applications.

**Fig. 1:**
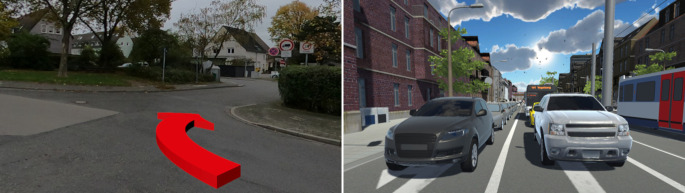
Augmented reality (AR) and virtual reality (VR) experiments. AR experiments (left) display virtual stimuli in a physical material environment. They often focus on applied research and have a very high ecological validity. VR experiments (right) display environments with fully virtual visual stimuli. They are characterized by a high control over visual and auditory stimuli and are therefore particularly suitable for basic research.

However, in the context of spatial experiments, the terms AR or VR can still be helpful to describe the focus of the experiment, even if an MR HMD is used. Often, AR experiments (see Fig. 1, left side) are designed for applied research such as testing different ways to communicate navigation information or providing information about specific spatial elements (Ayyanchira et al. 2022; Dickmann et al. 2021; Hou et al. 2015). They expose participants to physical material environments augmented with virtual elements and are particularly useful when the research approach requires a very high ecological validity or if the usability of an AR application is tested. Typical devices used for AR studies are the Microsoft HoloLens and the HTC Vive Pro. A weakness of AR experiments is the limited control over environmental parameters of the physical material environment presented such as lighting conditions, auditory stimuli, and temperature or visual distractors such as passers-by. These parameters can theoretically be controlled if the experiment is carried out in a laboratory. However, this undermines the key feature of AR experiments: letting participants perceive and interact with everyday environments augmented with virtual elements.

On the other hand, VR experiments are particularly suitable for basic research that requires a high degree of stimulus control. They can be easily carried out in laboratories to control parameters such as environmental temperature, olfactory stimuli, and sounds. As all of the visual stimuli provided are virtual, each participant can be exposed to the same lighting conditions as well as static and dynamic spatial stimuli (see Fig. 1, right side). However, the trade-off for the high experimental control is a lower ecological validity compared to AR experiments. Although modern game engines support the creation of highly realistic virtual environments, physical material environments cannot (yet) be replicated to an indistinguishable degree. Concerning available hardware, the range of compatible HMDs is much wider for VR experiments than for AR experiments including, for example, Oculus Rift, HTC Vive, HTC Vive Pro, Pimax 4K, Pimax 8K, Varjo XR4, Meta Quest Pro, Meta Quest 3, Valve Index, and many more.

### Immersion and the Feeling of Presence

As stated in the Introduction, a feeling of presence is a precondition for ecologically valid (realistic) behavior of participants within virtual or virtually augmented environments. In combination with software parameters, the occurrence of a feeling of presence is affected by a variety of hardware characteristics, the most important of which are discussed in the following sections. A system with these characteristics has the potential to create a feeling of presence and is labeled as being immersive (Slater et al. 1996). However, it is important to understand that the use of immersive hardware and software will not necessarily generate a feeling of presence within each participant. Participants must be willing and able to immerse themselves into the virtual or virtually augmented environments for a feeling of presence to occur (Baños et al. 2004). For example, a participant who is mentally preoccupied with another topic will be less likely to interact with a virtual environment or augmented virtual stimuli in an ecologically valid way.

In the context of spatial research, it is particularly spatial presence, i.e., the perceived sense of “being there” (Hruby 2019) within an environment, which is methodologically relevant. Spatial presence differs from related constructs such as social presence or self-presence, as it specifically refers to the cognitive and perceptual integration of spatial cues into a coherent environmental representation. For spatial cognition studies, presence is not merely desirable but functionally relevant, as it influences navigation behavior (Deggim 2024) and environmental memory encoding (Maneuvrier et al. 2020).

### General Hardware Specifications

Specifications of the XR hardware used in spatial studies can affect not only the occurrence of a feeling of presence, but also the findings obtained in general. Therefore, study reports should describe the hardware used in detail to ensure that the findings can be correctly interpreted and replicated by other researchers. Here, the most relevant hardware specifications of XR are described and their effects on immersion and spatial studies in general are discussed.

#### Depth Cues

Humans use a variety of cues to assess the distance of objects within their surroundings such as familiar object sizes (Hochberg and McAlister 1955), occlusion (Proffitt and Caudek 2003), and motion parallax (Rogers and Graham 1979). If a usually large familiar object visually appears to be very small, we can deduce that it is located far away. If object A is partially covered (occluded) by object B, then object A must be further away than object B. If during self-motion (or motion of an observed camera image), the relative movement of object A across the retina is slower than the relative movement of object B, object A is perceived to be further away.

These exemplary depth cues only require monocular visual input and can therefore be provided with any 2D screen. However, modern XR devices also enable stereoscopic perception of virtual stimuli. The stereoscopic perspective is provided by rendering two different images of virtual environments or stimuli from slightly different perspectives and displaying one of these images on a screen or reflective surface in front of the left eye and the other in front of the right eye. The discrepancies between the two images (binocular disparity) provide additional depth cues that are not available on traditional (single) monitors, as the discrepancy between the two images will be greater for closer virtual elements (Proffitt and Caudek 2003; Qian 1997).

Providing binocular disparity in modern HMDs greatly increases immersion compared to single 2D screens. However, even stereoscopic screens are not yet able to completely recreate depth perception in physical material environments. A remaining problem is the vergence–accommodation conflict, a conflict between an automatic reaction of the human eyes and the technical characteristics of current XR HMDs (Kramida 2016). When people fixate close objects, the eyes rotate inward (vergence). Simultaneously, eye muscles contract to change the shape of the lens (accommodation), which puts close objects into focus (Pielage et al. 2022). However, in most current XR HMDs, lenses (not to be confused with the lenses of the eyes) between the eyes and the screens are used to push the focal planes of the screens further away, as people cannot focus on screens located directly in front of their eyes (Martschinke et al. 2019). As the focal plane distance is fixed, focusing on virtual stimuli located closer or further away than this focal plane causes a conflict between the eye vergence and the eye accommodation and the stimuli appear to be blurry. This is especially the case for very close objects. Potential hardware solutions for the vergence–accommodation conflict are already being discussed and developed (cf. Kramida 2016; Zabels et al. 2019). For example, if the lenses of the HMD could dynamically adjust the focal plane to match the virtual distance of the fixated objects in real time, the vergence–accommodation conflict could be resolved. However, until such devices are commercially available and affordable, the use of very close virtual stimuli should be avoided if possible.

#### Spatial Tracking

One of the most prominent and relevant characteristics associated with immersive XR hardware is the tracking of head and body movements (Slater 2018). The tracking of movements is used to update the display of virtual stimuli to create the illusion of a permanent presence in a fixed location (Keil et al. 2019). This allows participants to change their visual perspective on virtual elements by rotating and moving their head or by moving through the available physical material space. Spatial tracking needs to be reliable, and the corresponding update of virtual stimuli needs to have a low latency. Otherwise, the resulting mismatch between the movements of the user and the perspective on virtual stimuli may not only deteriorate task performance and immersion but also cause VR sickness (also called “cybersickness”) with symptoms such as disorientation, headache, and nausea (Golding 2006; LaViola 2000; Wloka 1995).

**Fig. 2:**
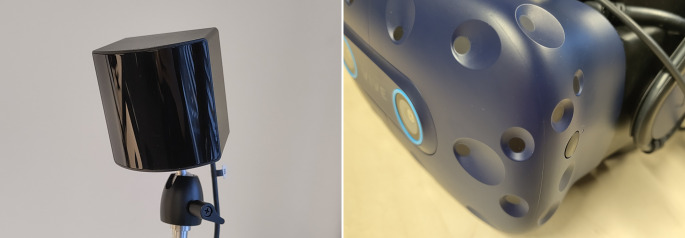
Infrared sensor-based tracking approach. Rotating lasers in the base stations (left) emit infrared light rays. Infrared sensors on the head-mounted displays (HMDs; right, in this case an HTC Vive Pro Eye) register these light rays. Based on the time differences when the rotating light rays hit specific infrared sensors, the position and rotation of the HMD can be calculated.

There are two main technical approaches to how user movements are tracked: infrared sensor-based and camera-based approaches. These two approaches are used in almost all commercially available HMDs. HMDs such as the HTC Vive, HTC Vive Pro, Valve Index, and Pimax 8K and the associated controllers use the infrared sensor-based approach developed by Valve Corporation. This approach requires one or several base stations (see Fig. 2, left side) to be installed around the user of the HMD. The base stations contain rotating lasers that emit infrared light rays (Valve Corporation 2025a). The HMDs and compatible controllers are equipped with multiple infrared sensors distributed over the surfaces of the devices (see Fig. 2, right side). Due to the rotation of the lasers, infrared rays will not hit all infrared sensors simultaneously but at different times. These time differences are used to calculate the position and orientation of the HMD and controllers (Keil et al. 2021b).

Using the SteamVR software, which is also provided by Valve Corporation, any compatible HMDs can be combined with any compatible controllers, even if they are not developed by the same manufacturer. Furthermore, using body trackers that can be attached to the ankles, elbows, or wrists enables limb tracking that can be used for realistic mirroring of user movements onto virtual avatars.

The infrared sensor-based approach developed by Valve Corporation provides a very high sub-millimeter precision with a very low latency (Caserman et al. 2019) as long as at least one base station is within the specified range and the straight line of sight between the base stations and the HMD’s (or controllers’) infrared sensors is not blocked by objects or the user’s body. Accuracy, however, depends on the spatial permanence of the base stations. If the base stations are moved, the orientation and location of the virtual environment and virtual stimuli relative to the physical material environment can shift and software recalibration of the tracked space is required.

However, the high precision and low latency of this tracking approach also come with trade-offs. First, the tracked space is limited to a maximum of 10 m × 10 m when four base stations are used simultaneously (Valve Corporation 2025a). With fewer base stations, the tracked space becomes smaller. This crucially limits the use of HMDs with base station tracking systems for studies requiring natural locomotion in large environments (see also Locomotion Methods section). Second, the infrared lasers can interfere with other devices that use infrared light. Third, adjusting or switching the tracked space requires time to install the base stations and recalibrate the HMD to the new positions of the base stations. Fourth, the base stations cannot be installed anywhere due to the required power supply.

**Fig. 3:**
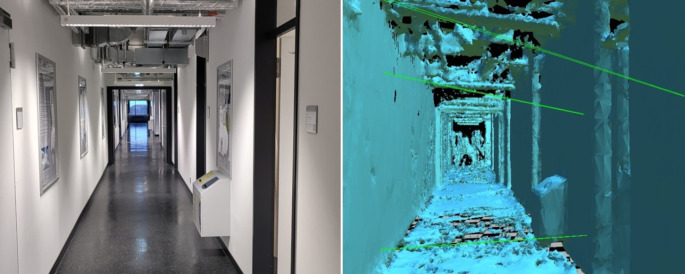
Camera-based tracking approach. Cameras located on the head-mounted display (HMD; in this example the Microsoft HoloLens) record the environment (left) and create a virtual spatial representation of the environment based on these recordings (right). The virtual spatial representation is then used to calculate the current location and rotation of the HMD and to update the display of virtual elements accordingly.

HMDs such as the Meta Quest series (Quest 2, Quest Pro, Quest 3, Quest 3S), Microsoft HoloLens, Pico Neo 3, and Apple Vision Pro use a camera-based tracking approach. Cameras located on the HMD (and in some cases on the associated controllers) are used to record the surroundings of the user (see Fig. 3). The recordings are then searched for objects that are used as spatial reference points. Based on the positions of these reference points, the relative position and rotation of the HMD can be calculated and used to update the display of virtual stimuli (Gourlay and Held 2017).

Compared to the infrared sensor-based tracking approach, camera-based tracking is spatially more flexible, as no base stations are required. Users can switch rooms and walk long distances while they are wearing the HMD without losing tracking, as the virtual model of the physical material environment is continuously updated (SLAM - simultaneous localization and mapping; cf. Jinyu et al. 2019). Thus, HMDs with camera-based tracking systems are useful for studies that require participants to walk through areas that are larger than the areas that can be covered with base stations (10 m × 10 m, Valve Corporation 2025a) such as navigation studies (e.g., Debandi et al. 2018).

The trade-off for the spatial flexibility of camera-based tracking is a poor accuracy and precision of the tracking system under specific circumstances. If the lighting conditions are suboptimal, for example, too bright, too dark, or if the environment contains highly reflective materials, then the cameras may not be able to identify the reference points required for spatial tracking (Eger Passos and Jung 2020). Furthermore, if HMDs with camera-based tracking are used in large empty areas such as a wide hall, the SLAM algorithm may have trouble identifying reference points that can be used to calculate their current position and rotation (Feigl et al. 2020).

Based on the described strengths and weaknesses of different spatial tracking approaches, it becomes clear that the selection of the HMD to be used for spatial research (and the corresponding spatial tracking approach) needs to fit the aims of the planned experiment. For example, an HMD with infrared-sensor-based tracking is recommended if very accurate tracking is a priority or the lighting conditions are suboptimal for a camera-based tracking approach. However, if natural locomotion across long distances is required, if the available space is often changed, or if the installation of base stations is not possible, an HMD with a camera-based tracking approach is recommended.

#### PC Peripheral or Standalone Device

Many HMDs such as the HTC Vive or the Valve Index are peripherals for personal computers (PCs). They contain screens used to present virtual environments and infrared sensors or cameras used to spatially track the users’ movements. However, a PC is required to process the sensor and camera data and to generate the images that are displayed on the screens of the HMD. This makes the use cases of these HMDs spatially less flexible, but it makes it possible to provide more graphic processing power and, consequently, render more graphically demanding virtual environments and display them at a higher frame rate. Furthermore, as the experiment file runs on the PC, the experimenter can control the experimental procedure using input from other peripherals such as a keyboard. For example, scenes can be switched, or specific stimuli can be inserted or moved based on keyboard input. The experimenter can also easily monitor participants’ behavior by mirroring the perspective of the HMD onto another monitor. This allows to ensure that the participants are attentive and complete the assigned task according to the provided instructions.

Usually, HMDs that act as PC peripherals have a wired connection to the PC. This connection not only transmits sensor and camera data as well as the images that are rendered on the PC, but it also provides power to the HMD. Consequently, the HMDs do not require a battery that would increase the weight of the device and needs to be recharged after a while of use.

HMDs with camera-based tracking (e.g., Microsoft HoloLens, Meta Quest series) are usually standalone devices. Standalone HMDs contain processors and batteries for energy supply. They do not require a PC for processing the spatial tracking data and rendering the displayed virtual stimuli or environments. Therefore, they offer greater freedom of movement than HMDs that act as PC peripherals. However, the installed battery increases the weight of an HMD and limits the usage time as it needs to be recharged after a while. This can be problematic if many participants are tested consecutively without pauses that can be used to recharge the device. In this case, it is recommended to choose an HMD with easily replaceable or even hot-swappable (changing components while the device is running) battery options. For example, third-party manufacturers offer secondary batteries for the Meta Quest 3 that are attached to the back of the head strap and can be hot swapped, as the primary battery is built into the device.

Furthermore, the visual quality of displayed virtual stimuli or environments is limited based on the available processing power of the device. Some standalone devices (e.g., Meta Quest series, Pico series) can be linked to a PC via a wireless local area network (WLAN) and the Steam Link app developed by Valve Corporations. This allows the HMDs to outsource image processing to the graphics processing unit (GPU) of the connected PC and increase the visual quality of displayed virtual stimuli or environments at the cost of spatial flexibility. However, due to the size of the transmitted data, using a WLAN with at least a 5-GHz band is recommended (Valve Corporation 2025b).

Standalone experiments also limit the ability of the experimenter to control the experiment procedure via keyboard input or to monitor the participants’ behavior by mirroring the perspective of the HMD to another monitor. Some standalone HMDs allow to connect Bluetooth peripherals and to stream the users’ perspective to a mobile app. However, the image quality is usually limited, and the streaming can negatively affect the performance of the HMDs, as it requires additional processing power.

In summary, HMDs acting as PC peripherals are a good choice if the experimenter needs to monitor the behavior of participants or if an experiment requires high graphical quality but does not require participants to move long distances. Standalone devices on the other hand are recommended if participants need to move longer distances using natural locomotion.

#### Obtrusiveness

In experiment design, obtrusiveness describes the potential of a measurement instrument, device, or procedure to distract participants from the experimental task or to negatively affect the ecological validity of the participants’ behavior (Kazdin 1979; Mellenbergh 2019). The reason for this undesired effect on participants’ behavior is that they are made aware of being part of an experiment and of their behavior being measured.

Several parameters can make participants aware they are wearing an HMD, consequently negatively affecting the feeling of presence within the virtual environments. This, in turn, may change the participants’ perception of, and interactions with, virtual stimuli.

It has already been mentioned that non-standalone HMDs usually have a wired connection to the PC. As reported by Kirakosian et al. (2021), half of their participants reported obtrusion induced by the HMD tethering. This HMD tethering limits how the participants can move. The moving distance is restricted to the length of the cable, and during rotations, participants need to be careful not to trip over the cable. The tripping issue can be resolved with cable management systems attached to the ceiling. However, the moving distance limitation remains. Furthermore, repeated rotations in one direction will result in a twisted cable that makes it more physically difficult for the participants to further rotate themselves in this direction. For some tethered HMDs, for example the HTC Vive Pro and the Valve Index, wireless adapters can be installed. This resolves the aforementioned issues but adds the requirement of regular recharging and increases the weight of the HMD.

Weight is another parameter that affects the obtrusiveness of an HMD. Heavy HMDs make fast head movements more difficult and can cause fatigue and neck pain (Ito et al. 2021). In particular, the built-in batteries in standalone HMDs increase the weight of the devices. However, in the context of potential neck pain, not only the weight of the HMD is relevant, but also the weight distribution of the device (Chihara and Seo 2018). Most devices have not only screens, cameras, and sensors built into the front of the device, but also batteries and processing units. Thus, the weight of the devices is often unevenly distributed, which must be actively compensated for by the neck muscles.

A (partial) solution for neck pain induced by unevenly distributed weight is the installation of counterweights. Some devices such as the HTC Vive XR Elite place the battery in the back of the head strap. As mentioned earlier, third-party hardware manufacturers offer alternative head straps with attachable power banks in the back for some HMDs such as the Meta Quest 3. The weight of these batteries shifts the center of mass further to the back and can thereby reduce the torque at the upper cervical spine (cf. Astrologo et al. 2024).

In the context of experimental design, it is desirable to reduce the impact of the aforementioned obtrusive parameters as much as possible. This allows participants to obtain a feeling of presence, ideally to forget that they are wearing an HMD, and to behave as naturally as possible. Therefore, if this is possible in the context of the investigated research question, using a wireless approach is recommended if participants need to move or rotate during the experiment. Furthermore, if participants need to wear the HMD for extended periods, using a lightweight HMD or adding counterweight batteries to the back of the head strap is recommended.

#### Field of View

The field of view (FOV) of an HMD affects how much of the displayed (virtual) environment users can see without moving or rotating their head. It affects the spatial awareness of the user, as peripheral visual stimuli may be overlooked if the FOV is very small (Keil et al. 2021b). As demonstrated by Lin et al. (2002), larger FOVs are associated with a stronger feeling of presence. Thus, an ideal FOV would match or exceed the natural human FOV. However, due to technical trade-offs associated with the lenses, screens, and processors used, no current commercial HMD covers the complete human FOV. Those HMDs that come close usually have strong image distortions around the image edges and a lower pixel density, as lenses with a stronger curvature are used to stretch the images displayed on the screens.

The size of the FOV depends not only on the lenses used, but also on eye relief, the distance of the lenses to the eyes (Sauer et al. 2022). In HMDs such as the HTC Vive and the Meta Quest 3, this distance can be adjusted in order to allow users to wear glasses while using the HMDs. However, the increased eye relief comes at the cost of a reduced FOV.

Some HMD manufacturers offer prescription lenses for HMDs that provide corrected-to-normal vision without wearing additional glasses. This makes it possible to decrease eye relief and, consequently, to increase the FOV. However, in the context of spatial studies, this is not a practical approach, as participants would require different lens strengths and switching between many different prescription lenses is expensive and time-consuming. Therefore, if it is necessary or desirable to include participants with impaired vision in the study sample who do not use contact lenses, it is recommended to use an HMD with adjustable eye relief and to ask participants to wear prescription lenses. The distance setting of the eye relief should then be standardized for all participants to ensure that variations of the FOV between participants are as small as possible. However, as eye relief is also affected by the individual shape of the face, small FOV differences between participants cannot be avoided (Mehrfard et al. 2019).

On the other hand, artificially reducing the FOV has been found to be an effective approach for reducing the occurrence and intensity of VR sickness (Adhanom et al. 2021; Fernandes and Feiner 2016). Thus, in experiments that are likely to cause VR sickness, it might be beneficial to accept a lower FOV and the associated reduced feeling of presence to improve the comfort of the participants. A prominent application example for an artificially reduced FOV is the use of artificial locomotion (see Locomotion Methods section). Especially in experiments with longer durations, these experimental designs tend to produce VR sickness. In these cases, FOV reductions may be applied either by using an HMD that already has a small FOV or by applying a software-based FOV reduction.

**Fig. 4:**
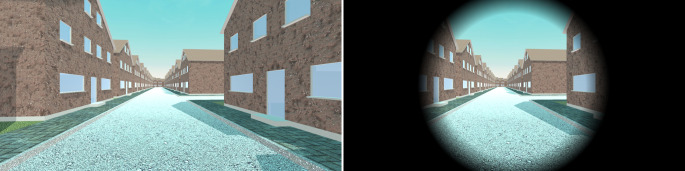
Tunneling vignette (right) used to reduce the field of view (FOV) of the displayed virtual environment (left). Although a large FOV is associated with greater spatial awareness and a higher feeling of presence, artificially reducing the FOV (e.g., during locomotion) can be a reasonable approach to reduce the risk of VR sickness.

Some VR software development kits (SDK) such as the XR Interaction Toolkit for Unity already provide a tunneling vignette as a built-in feature for reducing the FOV. The size and the appearance of the tunneling vignette can be linked to specific locomotion methods. For example, initiating a continuous artificial locomotion (see Locomotion Methods section) could automatically blacken the peripheral areas of the original FOV leaving only a circular central area of the original FOV visible (see Fig. 4). If the locomotion is stopped, the FOV may automatically return to the original size.

#### Image Clarity

Similar to televisions or monitors, the image clarity of HMDs primarily depends on the pixel density of the screens used. For televisions or monitors, the pixel density is usually specified as pixels per inch (PPI). Screens with a higher PPI can provide more visual information within a specific screen area. In the context of XR HMDs however, PPI is usually not a suitable measure for image quality. As the lenses between the user’s eyes and the screens are used to stretch the images of the usually rather small screens to a larger FOV, the PPI value of the screens does not match the observed pixel density. Therefore, the pixel density of XR HMDs is usually specified as pixels per degree (PPD).

**Fig. 5:**
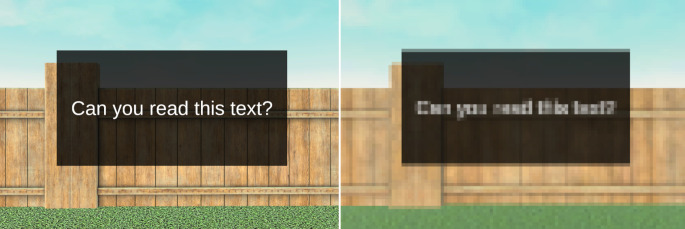
Extreme example of different pixel densities. Virtual environments displayed with a lower pixel density appear blurrier and text elements can become unreadable.

The pixel density of an HMD directly affects the user’s ability to perceive fine visual details such as text elements within the displayed virtual environments (see Fig. 5). This is especially the case if the visual elements are (virtually) further away. If the pixel density is too low, objects can appear blurry and the user can visually distinguish single pixels and the gaps between pixels (screen door effect; cf. Nguyen et al. 2020).

Considering its effect on image clarity, running spatial experiments on HMDs with a very high pixel density appears to be an obvious choice. However, a high pixel density also comes with a trade-off. Screens with a high pixel density require more processing power compared to displays of the same size but a lower pixel density. As the processing power (especially that of standalone HMDs) is limited, the additional processing requirements result in lower frame rates. This, in turn, can lead to VR sickness if a critical framerate threshold is crossed (Wang et al. 2023). Thus, when researchers choose an HMD model for spatial research, they must weigh performance against the presentation of fine details. Studies in which participants are already prone to VR sickness due to the study design (e.g., the use of artificial locomotion) should place more emphasis on a high frame rate at the cost of pixel density. If the virtual environment being displayed is minimalist and generally requires less processing power, there is more room for rendering on displays with a high pixel density.

## Software

Instead of having to build custom experiment software from the ground up, spatial researchers can draw on a wide range of software that supports the development of virtual environments as well as the integration and control of HMDs. Here, we address and compare the most prominent software packages and their most important features.

### Game Engines

Game engines are software that is aimed at supporting the development of 2D or 3D video games. However, they can also be used to quickly design highly immersive virtual environments for spatial studies. Modern game engines provide a great variety of features such as realistic physics simulations including artificial gravity and object collision calculation, lighting, 3D sound, and object interactions (Edler et al. 2019b; Ferworn et al. 2013; Indraprastha and Shinozaki 2009; Kersten et al. 2018).

Furthermore, spatial data can be applied to virtual terrains to design spatial representations of real spaces. Such representations can be used, for example, to evaluate the perception of different spatial planning scenarios. However, due to the variety of available geodata formats, intermediate transformation steps are often necessary. Various workflows have been described that enable the use of freely available spatial data in game engines (e.g., Edler et al. 2018; Hruby et al. 2019; Keil et al. 2021b; Krokos et al. 2019; Richter et al. 2022). Additionally, terrains and the arrangement of spatial elements such as street networks, buildings, or fauna can be procedurally generated (see Roglà Pujalt et al. 2017 or Vitacion and Liu 2019 for exemplary approaches). A great advantage of procedurally generated virtual environments is that the dimensions of the environments can be easily and massively increased once the system is set up.

Three of the most prominent game engines are Godot, Unreal Engine, and Unity. All three can be used free of charge for the development of non-commercial applications, but there are differences that should be considered when selecting the most suitable game engine.

Godot is free and open-source software (FOSS). The software can therefore be completely adapted to one’s own needs and freely be used even for commercial projects. However, both the commercial and the open-source aspects may be less relevant in the context of spatial research if it is not aimed at creating a commercial product or requires adjustments to the engine’s core components. Godot supports three different programming languages: GDScript, C#, and C++. Visual scripting, the ability to create game logic by creating and connecting graphical elements, is not officially supported by Godot, but it can be added via plugins.

The Unreal Engine is particularly suitable for extremely realistic visual applications due to its focus on the creation of AAA game titles. This focus appears to be reflected in the computing resource optimization. As reported by Kilijanek and Miłosz (2025), the Unreal Engine achieves high FPS (frames per second) stability in highly complex scenes, but is less efficient than Unity in scenes with lower complexity. When the Unreal Engine is used for the development of commercial applications, license fees may apply above a certain gross revenue threshold (Unreal Engine 2026). Game logic in the Unreal Engine is created in C++ or using visual scripting.

Unity is characterized by its very widespread use and the resulting mass of online documentation, tutorials, and community answers to technical questions. This makes the game engine particularly useful for beginners. Similar to the Unreal Engine, the free use of Unity is limited to non-commercial use or commercial use below a certain annual revenue (Unity 2026). Scripts for Unity are created in C# or via visual scripting.

All three engines provide asset stores that offer free or purchasable assets such as additional physics functions, textures, pre-made 3D objects, sounds, or visual effects. The use of available assets can greatly accelerate the development of virtual 3D environments, make it more cost-effective, and help to achieve a graphical quality that may otherwise not be possible with the available financial and time resources.

In summary, game engines help to create immersive and interactive virtual environments that spatial researchers could not realize without this software. The choice of the most suitable game engine depends on criteria such as potential commercialization, required functions, and degree of realism, as well as the developer’s prior experience including familiar programming languages.

### Software Development Kits for HMD Support

Thanks to XR software development kits (SDKs) such as SteamVR, OpenXR, or Meta XR All-in-One SDK, virtual 3D environments created in game engines can easily be made compatible with XR hardware.

The SDKs ensure that the HMDs and associated controllers used are recognized and can interact with the VR application. Furthermore, XR SDKs typically provide templates and demo scenes that facilitate and demonstrate the implementation of standard features such as mirroring the movements of the HMD and controllers within the virtual environment, artificial locomotion (see Locomotion Methods section), and interaction with virtual objects.

SDKs for XR support are available for various game engines. Some are integrated into the game engines, while others are offered by HMD developers. In most cases where access to the specific features of a particular HMD model is not required, using OpenXR is recommended. OpenXR is an open industry standard that simplifies the implementation of support for various HMDs and controllers, even from different companies, within a single application.

### Experiment Toolkits

While game engines and SDKs provide all the standard features needed to build virtual environments and make them VR-ready, spatial experiments typically require additional features such as stimulus randomization, recording demographic and experimental data, or implementing questionnaires, for which the standard functions of game engines and SDKs are not designed. This gap is filled by experiment toolkits, which can be integrated into game engine projects. The specific experiment toolkits that can be used depend on the game engine being employed.

The focus and range of functions included can vary greatly between different experiment toolkits. For example, the PLUME toolbox for Unity is aimed at supporting the recording of XR-related behavioral and physiological data, including the creation of heatmaps of gaze data and interacted virtual objects, as well as recording and replaying experimental sessions (Javerliat et al. 2024). The Unity Experiment Toolbox (UNEXT) provides functions for recording demographic variables and experiment data, complex scene loops, randomizing the display of stimuli, and creating questionnaires (Keil et al. 2026). The StudyFramework for Unreal Engine supports condition randomization, recording of positional data and events with timestamps, and provides an experimenter view that communicates information about the current experiment status (Ehret et al. 2024).

These toolkits are only examples of the large number of available options and their range of functions, which have been chosen based on their broad approach that can be used for many different experimental designs. If a spatial experiment follows a very specific research approach, it can be useful to research whether there is a toolkit that specializes in this specific approach and can facilitate its technical development.

Taken together, the current XR landscape offers a wide range of hardware and software options for spatial research. Depending on the purpose of a spatial study, available resources, and the developers’ prior knowledge, different game engines, SDKs, and experiment toolkits can be used.

## Experiment Design

When using XR hardware in spatial studies, classic approaches to experimental design must be reconsidered. Some commonly used approaches, such as fully standardizing the stimulus display in monitor-based studies, do not work in XR applications. In these cases, approximate solutions are necessary. In other cases, the use of XR hardware opens up new possibilities that can be used for innovative research approaches. Here, we describe relevant unique features of the design of spatial studies with XR hardware and their implications.

### Locomotion Methods

In AR applications where the physical material environment is augmented with virtual objects, only natural locomotion can be used. This means that if people want to move around a static virtual object, they must actually perform this movement in the physical material environment. In VR environments where the physical material environment is not visible, a wider range of locomotion methods can be used.

Similar to AR applications, natural locomotion (also called “room-scale locomotion”) is possible in VR applications. Depending on the HMD used and the integrated spatial tracking system, the locomotion space is either limited to a small tracked area or, with certain restrictions (based on spatial reference cues and lighting), can be extended to large areas of the available space in the physical material environment (see Spatial Tracking section). When using natural locomotion, a system is typically employed that visualizes the boundaries of the available free space in the physical material environment using a grid system (also called “chaperone system”) as soon as the user gets too close to these boundaries. This helps to avoid collisions with walls or objects. Natural locomotion is characterized in particular by a very low susceptibility to VR sickness, since all movements of the subjects in physical material space are mirrored in the virtual environment. Therefore, there is no discrepancy between proprioceptive (e.g., muscles and tendons; cf. Dietz 2002) and vestibular (inner ear; cf. Angelaki and Cullen 2008) body feedback about movement in space and the visually perceived locomotion (Langbehn et al. 2017). Natural locomotion has also been associated with a strong feeling of presence (Usoh et al. 1999). The use of an XR SDK usually automatically adds a template for natural locomotion, as the spatial tracking and mirroring of the HMD’s movement, which automatically ensures the ability to use natural locomotion, is the most basic requirement of VR applications.

However, the use of natural locomotion has one important disadvantage: it limits the explorable space in the virtual environment to the available free space in the physical material environment. As some spatial studies depend on participants exploring extensive virtual environments, other—artificial—locomotion methods can be required.

**Fig. 6:**
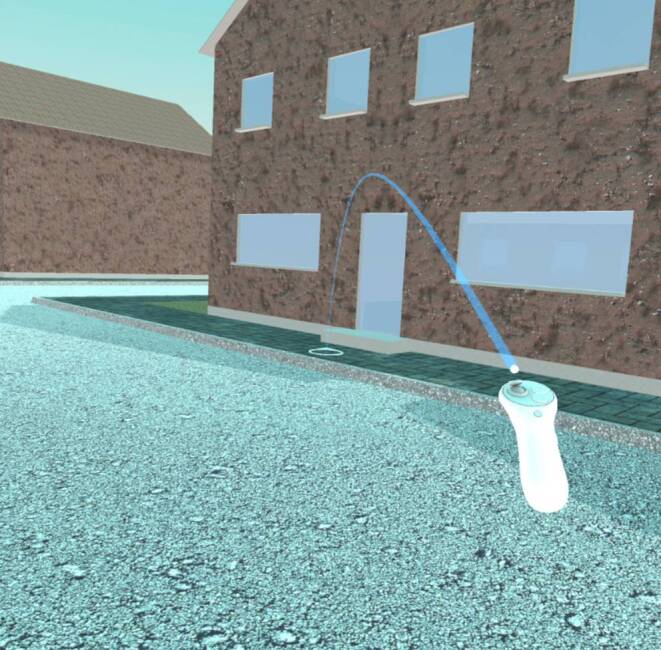
Teleportation system included in the OpenXR SDK for Unity. By aiming at a location of a surface that has the *TeleportationArea* script attached to it and pressing the assigned button, the user can instantly teleport to the selected location.

Teleportation is an artificial locomotion method with low susceptibility to VR sickness (Langbehn et al. 2018). By aiming a controller at a desired target location, usually supported by a virtual beam or arc of light, and pressing a button on the controller, the user is instantly transported to the selected target point (see Fig. 6) and thereby travel distances that are larger than the available physical material space. If the experiment design requires a limitation of the possible visual perspectives and the traversable space, it is possible to either define only specific surfaces as potential teleportation targets or to set fixed teleportation anchors (point objects) that can be selected as teleportation targets. By using teleportation repeatedly, participants can cover very long distances in a short time.

Since there is no artificially induced optic flow during teleportation, there is also no discrepancy between proprioceptive, vestibular, and visual locomotion feedback that could induce VR sickness. Teleportation is often combined with natural locomotion where teleportation can be used for movements over long distances, while natural locomotion is used for rotation and short-range movements (Cherep et al. 2020). Since instantaneous movement between two points without optic flow differs significantly from natural locomotion, teleportation can negatively impact the feeling of presence. Users are thus reminded that they are not perceiving a real environment, which may affect the ecological validity of acquired experimental results. Furthermore, findings of Cherep et al. (2020) indicate that the lack of translational self-motion cues can negatively affect performance in spatial tasks such as path integration.

A second commonly used artificial locomotion method in VR is continuous locomotion. By using controller input such as pressing a touchpad or a thumbstick into the desired walking direction, the location of the user within the virtual environment is continuously translated into the selected direction. The continuous locomotion speed can be freely defined. Either a fixed value can be chosen, or the user can be given the option to dynamically choose the speed up to the specified maximum value by pressing a specific position of a touchpad or only partially pressing a thumbstick into the desired direction. Depending on the requirements of a spatial experiment, movement in certain directions can be blocked, so that, for example, only forward movement is possible (e.g., Keil et al. 2021a).

Contrary to teleportation, continuous locomotion provides optic flow that helps users perceive they are moving through the virtual environment. Similar to traditional monitor-based spatial studies, the optic flow information can be used to estimate traveled distances, memorize traveled paths, and perform path integration tasks (Cockton and Korhonen 2003; Frenz et al. 2007; Korte et al. 2025). However, the non-natural way of locomotion can negatively affect the feeling of presence and thereby the ecological validity of the experimental results obtained. Furthermore, continuous locomotion is characterized by high susceptibility to VR sickness, as the perceived optic flow does not match the proprioceptive and vestibular feedback (Langbehn et al. 2018). A partial solution for the occurrence of VR sickness is to reduce the FOV during continuous locomotion (see Field of View section) or to select a very low maximum locomotion speed. Nevertheless, when using artificial locomotion, the experiment duration should be kept as short as possible, and it should be expected that some test participants may need to abort the experiment due to discomfort.

A third approach to artificial locomotion, intended to reduce the disadvantages of continuous locomotion, is the use of omnidirectional treadmills. These devices usually consist of a horizontally rotatable hip lock for the user and a wok-like bowl under the feet, through which the user can slide with their feet. Based on the user’s orientation and the speed of their foot movements, the user is translated forward through the virtual environment. Unlike controller-based continuous locomotion, this method of locomotion offers not only optic flow cues but also proprioceptive feedback. Despite this additional body feedback, several studies have reported that symptoms of VR sickness can occur when using omnidirectional treadmills (Lohman and Turchet 2022; Wehden et al. 2021). This could be because these devices provide proprioceptive but not vestibular feedback associated with forward locomotion, as the users are walking “in place.”

Which of the available methods of locomotion is most suitable depends on the specific characteristics, requirements, and goals of the planned spatial experiment. Particularly in spatial cognition studies, it must be addressed that the chosen method of locomotion may in some cases influence spatial perception. For example, distance estimations using artificial locomotion methods in VR were found to be underestimated with particularly high underestimations when teleporting was used (Keil et al. 2021a). On the other hand, Xu et al. (2017) found no effect of locomotion technique in VR on object location memory. Still, potential effects of missing optic flow, proprioceptive and vestibular feedback on spatial perception need to be considered.

**Fig. 7:**
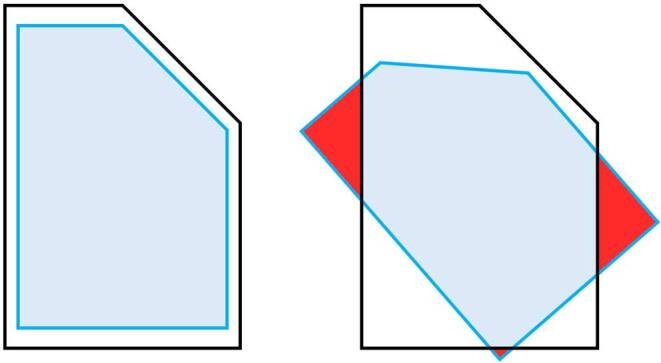
Mismatch between available space in the virtual and physical material environment. If the virtual environment is designed to match the available space in the physical material environment, artificial locomotion techniques need to be disabled to prevent a mismatch between the two environments.

Other parameters that need to be considered when choosing a locomotion technique are the size of the available experimental area, the size of the virtual environment, and the desired sample composition. If the distances that need to be traveled in the virtual environment are larger than the available space in the physical material environment, artificial locomotion (potentially in combination with natural locomotion) needs to be used (Kelly et al. 2020). However, if the virtual environment is optimized for natural locomotion by fitting the dimensions to the available space and barriers within the physical material world, artificial locomotion techniques should be disabled. Otherwise, any artificial locomotion-based change of location or orientation would result in a mismatch between the traversable virtual and physical material space (see Fig. 7). In these cases, participants could walk into a wall or, when a chaperone system informs them about physical barriers, only parts of the virtual environment could be explored.

If the study sample is targeted at a specific age group, differences in the susceptibility to VR sickness need to be accounted for. As young people have been found to be more susceptible to VR sickness (cf. Saredakis et al. 2020), the use of continuous locomotion should be avoided where possible, if the spatial study aims to examine adolescents.

### Stimulus Control

In monitor-based spatial experiment design, researchers have full control over stimulus presentation as long as the participants are not able to change their visual perspective, for example, via controller or keyboard input. However, experimenters cannot control to which areas of the presented stimuli participants direct their visual attention.

Providing ways for changing the visual perspective by moving or rotating a virtual camera allows participants to explore virtual environments more naturally. Allowing self-controlled movement is essential for many spatial studies such as evaluating spatial planning scenarios or investigating spatial behavior and memory. However, it limits the ability of the experimenter to control which stimuli are presented at a specific moment, and to investigate in a controlled manner how specific stimuli influence spatial perception and behavior.

**Fig. 8:**
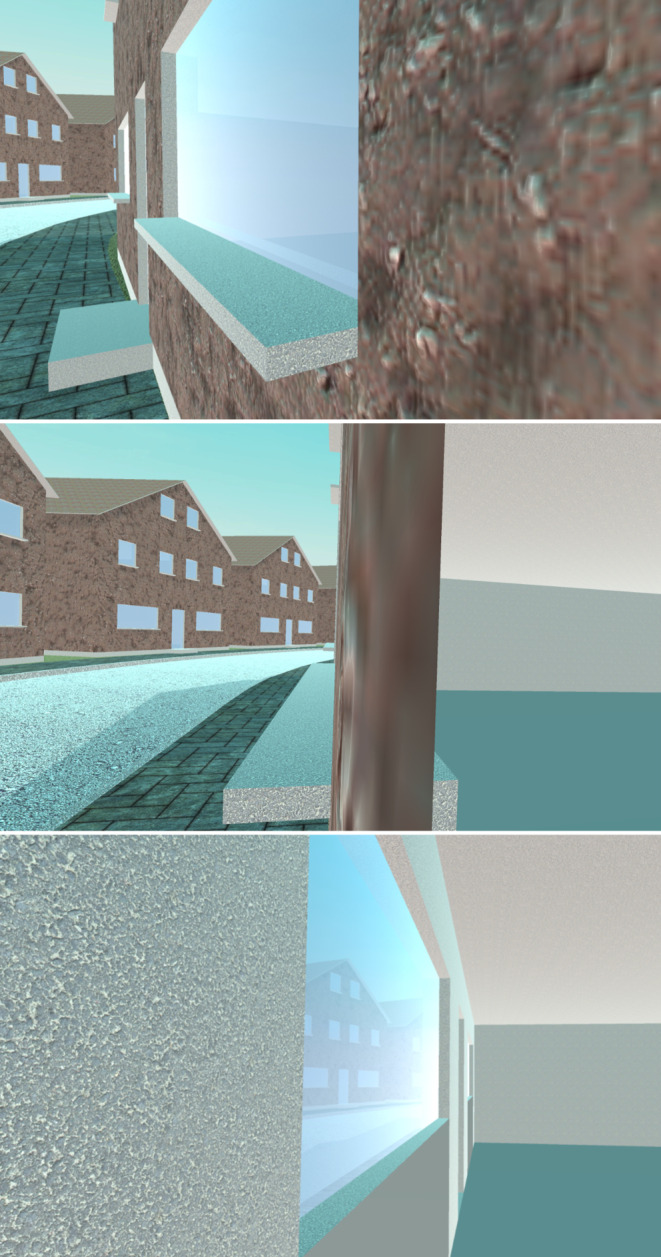
Limited control over the visual perspectives taken using extended reality (XR) hardware. Even if the artificial locomotion in virtual environments is limited to meaningful areas, participants may take on undesired visual perspectives by physically moving their bodies or heads, as these movements are mirrored within the virtual environment. By doing this, participants could for example “stick their heads through a wall”.

Displaying virtual stimuli using XR hardware further complicates experimental control over stimuli, as the visual perspective of the participants is subject to additional degrees of freedom. Using head and body movements, which are mirrored into virtual environments, participants can change their visual perspective in ways that may be unwanted from the perspective of the researcher. As head and body movements function independently of predefined limitations for artificial locomotion such as assigned teleportation areas, participants can even put their heads through walls or other “massive” objects (see Fig. 8). Movement areas should therefore be planned in advance in such a way that participants do not get too close to such collision objects. Otherwise, gathered data may need to be filtered retrospectively to ensure that unwanted behavior does not distort the findings. The degrees of freedom in perspective can at least be limited if the participants are asked to stand still in one place or sit on a chair. However, this comes at the expense of interaction with the virtual environment that is as natural as possible and, as a consequence, at the expense of reduced presence and ecological validity of the experimental results.

Still, compared to experiments carried out in physical material environments, using virtual environments and XR hardware provides highly superior stimulus control including the presence of distractors and general environmental conditions (Minderer and Harvey 2016). Especially for outdoor scenarios, the ability to control the appearance of passersby, daylight conditions, weather, environmental temperature, or ambient sounds and odors in virtual environments enables data to be collected that is much more comparable between participants and recording times.

### Directing Visual Attention

Many experimental designs studying human participants require a certain amount of control over where participants are looking. Cognitive studies displayed on 2D monitors often use fixation crosses at the beginning of each trial to direct visual attention to the center of the screen or a different relevant screen location before the next stimulus is presented (e.g., Korte et al. 2023; Wieser et al. 2009). The standardization of the initial gaze direction is of particular relevance when eye movements are recorded. If the initial gaze of all participants is directed toward the same stimulus area, following eye movements can be better assigned to stimulus characteristics such as visual salience (cf. Keil 2021; Wenczel et al. 2017) or general eye movement patterns such as following the learned reading direction (cf. Afsari et al. 2016).

**Fig. 9:**
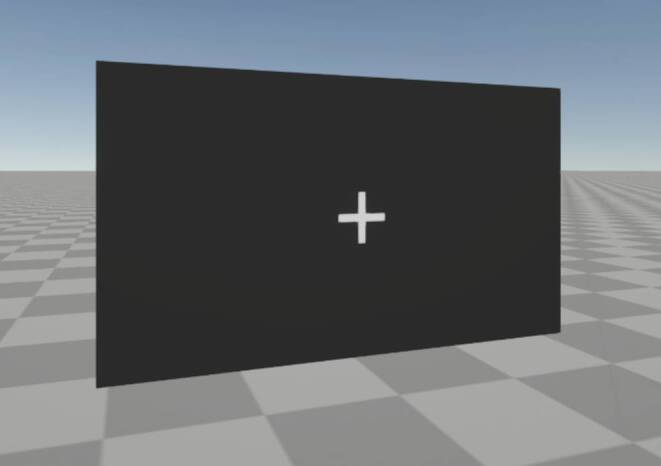
Display of a fixation cross in a virtual reality (VR)-based virtual environment. Fixation crosses can be placed on a canvas within the virtual environment. However, as participants can change their visual perspective by moving and rotating their head, it cannot be ensured that they direct their visual attention toward the fixation cross.

In VR-based studies, fixation crosses can be placed on a canvas located anywhere in the virtual environment (see Fig. 9). However, this does not ensure that participants actually direct their visual attention towards the direction of the fixation cross even if they are instructed to do so. If other elements of the virtual environment are still visible, these elements may also attract visual attention. Fixation crosses may even be missed if participants look in the opposite direction when they appear.

A possibility to address this issue is to rotate the player toward the desired direction via code when a new scene is loaded or a new stimulus is displayed. However, although this prevents participants from missing the fixation cross, it still does not guarantee that they actually fixate the fixation cross. Additionally, as mentioned earlier, artificially rotating the visual orientation of participants is not recommended if natural locomotion is used and the virtual environment is designed to fit into the available physical material space.

**Fig. 10:**
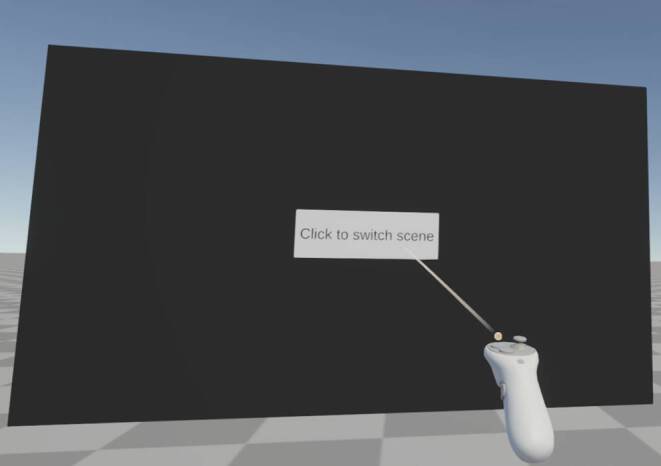
Alternative to fixation cross. To ensure that participants actually look into the required direction, they can be instructed to press a button on a canvas using controller input.

An alternative approach to the traditional fixation cross is to place a button on a canvas that participants need to press using controller input to trigger a specified action such as a scene change or the display of the next stimulus (see Fig. 10).

### Eye Tracking

Some HMDs, such as the HTC Vive Pro Eye and the Meta Quest Pro, have built-in eye trackers and can capture fixations on displayed virtual objects. Although the direction of visual attention does not match the fixated spatial objects in each case, fixation data provide a good estimation of the visual objects being cognitively processed (Just and Carpenter 1976; Poole and Ball 2006). Thus, based on recorded fixations, spatial behavior or physiological responses can be associated with the cognitive processing of specific spatial objects. Furthermore, differences in object saliences based on spatial design changes can be derived (Keil 2021).

In contrast to the assessment of eye fixations on physical material objects, detecting fixations directed at virtual objects does not require manually defining areas of interests that match the investigated spatial objects on a frame-by-frame basis. Instead, a function that casts rays out of the virtual eye positions in the viewing direction can be used to detect collisions with objects in the virtual environment. Based on the detected collisions and specified fixation filters, fixations on objects can be calculated from any viewing direction.

One minor weakness of the raycasting approach (which also applies to eye tracking in physical material environments) is that if transparent objects like windows are in the viewing direction, fixations are assigned to the window surface, even if objects behind the window are actually being fixated. This occurs because the first collision of the cast ray with an object is evaluated. This problem could theoretically be corrected if eye vergence information could be used to calculate the distance of the fixated object. However, this approach is practically difficult to implement, since even large differences in distance result in only small differences in eye vergence for more distant objects. As an alternative solution, if transparent surfaces are to be excluded from the detection of fixations, they can also be assigned to a rendering layer that is ignored by the raycasting function. In this case, fixations on objects located behind these surfaces will be detected, but not fixations on the surfaces themselves.

**Fig. 11:**
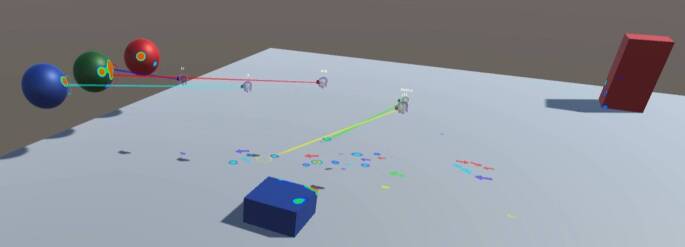
Investigating eye fixations in virtual environments with Tobii Pro VR Analytics (software discontinued). The eye tracking software tool makes it possible to track user movements through the virtual environment, gather and visualize real-time gaze data, replay user behavior with several participants displayed simultaneously, and generate 3D fixation heatmaps for specified participants and time frames. This allows movement and fixation patterns across participants to be easily identified.

A remaining obstacle to recording and analyzing eye movement data associated with the perception of virtual stimuli is the availability of software tools. With few exceptions (see Fig. 11), existing software solutions are not geared toward research purposes, but rather toward gaming (eye-based interaction or foveated rendering) or marketing (simple salience evaluations). Therefore, depending on the HMD used, the game engine used, and the research purpose, it may be necessary to develop a custom software solution for recording and evaluating fixations.

In summary, the use of XR hardware requires substantial modifications to the experimental design compared to monitor-based studies or studies carried out in physical material environments. Based on the aim of a study, a locomotion method must be selected that enables the required freedom of movement but restricts participants from undesired visual perspectives. Furthermore, the experimental design must consider the occurrence of VR sickness and the ability to ensure that participants perceive the intended visual stimuli.

## Conclusion

Utilizing extended reality (XR) hardware provides innovative opportunities for spatial experiment designs. Whereas augmented reality (AR) studies are often aimed at evaluating the effectiveness, efficiency, and overall experience of AR visualizations, virtual reality (VR) studies fill the gap between monitor-based spatial experiment design and studies carried out in physical material environments. They provide a more immersive and natural experience compared to monitor-based studies and acquired findings are ecologically more valid. On the other hand, they provide better experimental control compared to experiments carried out in physical material environments, and the choice of experimental environments is not limited by parameters such as remoteness, environmental hazards, or even non-existence. Using an XR head-mounted display (HMD), participants could virtually experience any location on Earth, the surface of another planet, the magma chamber of an active volcano, or an infinite empty plane. Furthermore, using XR hardware facilitates the implementation of eye tracking and the evaluation of gaze data, as information about fixated virtual objects can be automatically retrieved and processed, even if these objects are perceived from varying visual perspectives.

Beyond offering new technical possibilities, XR fundamentally reshapes methodological considerations in spatial research. Decisions regarding hardware specifications, tracking approaches, locomotion techniques, or rendering quality are not merely technical optimizations—they actively shape perception, interaction, and ultimately the empirical findings derived from an experiment. Consequently, the integration of XR into spatial studies requires a shift toward technologically reflexive experimental design, in which methodological transparency and justification of design choices become central components of scientific rigor. Given the rapid succession with which new XR hardware and software are made available and the sometimes associated short lifespan (discontinued products), long-term replicability of spatial experiments cannot always be guaranteed. However, a detailed description of the hardware and software used, the motivation behind the decisions, and the availability of self-developed software can improve replicability.

Designing AR or VR experiments confronts experimenters with new requirements, challenges, and trade-offs. Numerous decisions to be made such as choosing an HMD, a game engine, additional software solutions, and available locomotion methods are not trivial and directly affect the resulting experiment design, the users’ experience, and the validity of the acquired data. Unfortunately, there is no ideal approach that can be applied to any VR or AR experiment. Instead, the advantages and disadvantages of specific hardware, software, or experiment design decisions need to be carefully weighed in the context of the specific purpose of the experiment (see Table 1). Furthermore, despite the extensive support provided by numerous software solutions such as game engines, software development kits (SDKs), and toolboxes as well as online documentation and support forums, the development of spatial studies utilizing XR hardware still requires some basic programming experience and willingness to engage with the subject matter.

**Table 1:** Exemplary design choices for spatial experiments with XR hardware with associated benefits, issues, and mitigation strategies. These design choices are merely illustrative of the great variety of potential combinations of hardware and software parameters. However, they provide initial indications of the consequences associated with the selection of certain experimental parameters.

General experimental setting	Experimental design parameters	Benefits	Issues	Mitigation strategies and alternative design choices
High-control small-scale VR	Tethered HMD, natural locomotion, carried out in a laboratory	Full control over visual stimuli and environmental parameters, processing power not limited by HMD, highly immersive locomotion	Limited locomotion space, obtrusion of tethering	Wireless adapter for HMD, cable management systems, artificial locomotion method
Exploratory large-scale VR	Standalone HMD, natural locomotion, carried out in an outdoor environment	Full control over visual stimuli, highly immersive locomotion across larger distances	Processing power limited by HMD, VR-sickness induced by low frame rates, experiment duration limited by battery, limited control over environmental parameters (temperature, noise)	Limiting visual details of the VR environment, hot-swappable external batteries
High-control large-scale VR	Tethered HMD, continuous (controller-based) locomotion, carried out in a laboratory	Full control over visual stimuli and environmental parameters, processing power not limited by HMD, locomotion across larger distances	Obtrusion of tethering, locomotion less immersive, risk of VR-sickness	Wireless adapter for HMD, cable management systems, field of view reduction, limiting experiment duration
Exploratory large-scale AR	Standalone HMD, natural locomotion, carried out in an outdoor environment	Highly immersive locomotion across larger distances, high ecological validity	Processing power limited by HMD, experiment duration limited by battery, limited control over environmental parameters (temperature, noise), tracking accuracy depends on lighting conditions and available reference points	Hot-swappable external batteries, use of large-scale indoor laboratory

*AR* augmented reality, *HMD* head-mounted display, *VR* virtual reality, *XR* extended reality

Here, we provided an overview of a variety of important experimental parameters associated with the use of XR hardware and their impact on the design of spatial studies. This overview may help spatial researchers to transition their experimental designs from monitor- or physical material environment-based approaches toward immersive AR or VR approaches that provide a middle ground between immersion and experimental control, as well as a maximum flexibility of the presented visual stimuli. By utilizing the potentials of XR hardware, innovative spatial research designs become feasible.
